# Suggestions for a New Policy

**Published:** 1919-12-06

**Authors:** 


### THE RECONSTRUCTION OF ADMINISTRATION.

### Suggestions for a New Policy.

The great changes which are taking place in the voluntary hospital system have begun to affect administration; and the reconstruction of that is as necessary as the reconstruction of buildings or finance. In fact the three are bound with one another. If we begin by considering the shortage of beds, and the need for increasing their number, the three problems can be considered in turn. The first thing that often happens to a hospital with a scandalously long waiting-list is a demand for the space occupied by the secretarial department. To place beds in its sacred precincts, the office has to be moved from the hospital itself to the nearest house outside which can be adapted for the purpose. Where the house governor is already living out this, however regrettable, is comparatively easy. But there can be little doubt that the house governor, whom we may assume to be married, should live in the hospital because he is the hub on which the whole administration turns. If this is true of the house governor's personal quarters, it is also true of his department. That should be where it is wanted—namely, at the centre. To move him out, if he is resident, and to remove his office, if he is not, is a serious step, which can be accepted only as a provisional and temporary measure. But it must be regarded as the price exacted for past neglect to provide the necessary number of beds, to which neglect the disturbance is primarily due. When matters have reached the crux which necessitates the invasion of his quarters, the secretary will be hampered and lose his free play at the very moment when he most needs escape from the detail of routine in order to devote himself to raising the large funds which alone can provide the necessary beds, maintain them, and once more place the hospital and its administration on a proper basis. A reform of his department becomes essential, and is doubly pressing when his accommodation is commandeered.

### Refinement without Complication.

The problem for the house governor is to refine his organisation without complicating it, and to do this he may divide the work in the following way. If he divides his department into two sections, he will place the routine work of correspondence, meetings, and minutes in the hands of his clerks, and devote another section to finance. But this crude division is meaningless unless the question of finance is understood to involve separately the spending and the raising of money. To spend money wisely the treasurer must have a committee in charge of investments, estimates, and the like; while the secretary must supervise, or have under him a supervisor of (*a*) stores and their allotment, (*b*) the waste in little things which can become a very heavy item at the end of a financial year. For the second of his duties, the supervisor will need to be constantly about the hospital, to prevent waste, to report on the need for repairs, and, arising from these duties, to look after the discipline of the junior staff and the porters. Whether the man entrusted under the secretary with this work can combine it with the duties of steward or stores clerk is a practical rather than a theoretical question. The right man, with the house governor behind him to whom he would report any important matter, could do so. The wrong man would not save the house governor's time, but create mischief. None of these questions can be decided without reference to the human material on which each house governor has to rely. So much briefly for the spending department.

### The Raising of Money.

In the writer's view, the raising of money should not be in the hands of the treasurer and the financial committee, except in the rare case when the treasurer happens to be a man of the type of Barnardo or the late Colonel E. M. Bruce-Vaughan of Cardiff. The treasurer and the financial committee should report the financial requirements; the drafting of appeals should be left to the secretary's department, if only because that department deals with correspondence, which, in practice, provides much of the best material for appeals. It is hardly too much to say that no letter should leave the secretary's office which does not have *the effect of* an appeal. The word "appeal" should be shunned; it stops the well-spring of sympathy. The aim is to produce the effect of which the ordinary appeal is but the caricature. All powerful influence is indirect. It is as true of appeals as of children, that those who ask do not have; while those who make other people laugh or cry very often get what they want. The appeals, then, should be in the hands of the secretary, because he receives the letters which acquaint him with that which outside people want.

### Medical Superintendents.

It is because a modern house governor has to be a man of education and resource that the American plan of preferring medical superintendents has found favour. The plan guarantees a degree of education; nothing but its fruits can guarantee resource. On the other hand, how can a layman as the administrative head of a large hospital display his quality or exercise his gifts if he is smothered in detail? He must be given the chance which is given as a matter of course to a medical superintendent, the chance to be the man of ideas as well as the man of detail. Relieve him from this last by reconstructing his department on the lines suggested tentatively here, and, if he is the right man, not only will the devolution of duties improve the standard of daily work, but he will be free to tackle the larger problems of finance and reconstruction, for which leisure and free play are as necessary as character.

